# Assessing the spatial influence of deforestation on malaria incidence in Pará State, Amazon region, Brazil, 2008-2019

**DOI:** 10.1590/0074-02760240143

**Published:** 2025-06-13

**Authors:** Carla Gisele Ribeiro Garcia, Beatriz C Ribeiro, Alcinês S Souza, Lilian Jéssica P Lima, Marinete M Póvoa, Gabriel Z Laporta, Maristela G Cunha

**Affiliations:** 1 Universidade Federal do Pará, Instituto de Ciências Biológicas, Laboratório de Microbiologia e Imunologia, Belém, PA, Brasil; 2 Secretaria de Estado da Saúde do Pará, Belém, PA, Brasil; 3 Ministério da Saúde, Instituto Evandro Chagas, Seção de Parasitologia, Ananindeua, PA, Brasil; 4 Centro Universitário Faculdade de Medicina do ABC, Pós-Graduação e Pesquisa, Santo André, SP, Brasil

**Keywords:** Amazonian ecosystem, Brazil, conservation of natural resources, environment and public health, incidence, malaria, poverty

## Abstract

**BACKGROUND:**

Malaria transmission is prevalent in tropical regions and is heavily influenced by environmental factors such as deforestation, which is particularly significant in the Brazilian Amazon, especially in Pará State.

**OBJECTIVE:**

This study aimed to assess the relationship between deforestation indicators and malaria incidence across all 144 municipalities in Pará.

**METHODS:**

Using municipal-level data from 2008 to 2019, the study applied geographically weighted regression (GWR) to analyse spatial relationships between malaria incidence and deforestation metrics. These metrics included forest cover loss from the previous year, pastureland, forest cover, fragmentation, urbanisation, and water levels, analysed over three distinct 4-year periods. The study also incorporated poverty levels to examine their influence on municipalities with high malaria risk.

**FINDINGS:**

During the study period, the total deforested area in Pará was 30,000 km^2^, with 679,846 malaria cases reported. Malaria incidence rates varied across municipalities, with stable rates in high-risk areas, and were linked to pastureland, forest loss, fragmentation, and forest cover. The GWR models effectively captured spatial heterogeneity in these interactions.

**MAIN CONCLUSIONS:**

Malaria incidence was associated with areas of Pará State experiencing significant forest loss and fragmentation, indicating that changes in forest composition and configuration influence malaria risk.

Malaria is a disease caused by parasites of the *Plasmodium* genus, which are transmitted by anopheline mosquitoes. Five species of this genus can infect humans, with *Plasmodium falciparum* and *P. vivax* being the most prevalent.^1^ ^,^ ^2^ ^,^ ^3^ Geographically, *P. vivax* predominantly affects populations in various regions, including Asia, Oceania, and South America. This includes a substantial area in northern Brazil, the Amazon region.^4^ ^,^ ^5^ ^,^ ^6^ ^,6)^

Malaria transmission is primarily confined to tropical zones, where environmental factors influence parasite transmission. Notably, deforestation holds particular significance because of its increase in tropical areas, specifically in the Brazilian Amazon region.^7^ ^,^ ^8^ ^,^ ^9^ As a result, ecological factors play a role in regulating the species composition of mosquito populations, influencing both the number and types of malaria vectors.^10^ ^,^ ^11^

The interplay between malaria transmission, forest cover, and deforestation is intricate, directly affecting all transmission components, namely the vector, host, and the environment.^8^

The incidence of malaria is contingent upon environmental factors that facilitate the proliferation of vector mosquitoes, including their adaptation to climate, altitude, and vegetation.^9^ ^,^ ^11^ ^,^ ^12^ Alterations to the environment resulting from economic activities such as land use^13^ ^,^ ^14^ can contribute to the proliferation of vectors, human exposure to infected mosquitoes, mosquito biting rates, and ultimately, the incidence of malaria.^14^ ^,^ ^15^ Malaria transmission is further influenced by the living conditions of the population, exhibiting a strong association with poverty and economic activities that contribute to deforestation, such as agriculture and mining, which are common in the Brazilian Amazon.^16^ ^,^ ^17^ ^,^ ^18^ ^,^ ^19^ Previous studies have indicated that deforestation may increase the incidence of malaria.^8^ ^,^ ^9^ ^,^ ^11^ ^,^ ^12^ Nevertheless, other reports emphasise that the impact of this factor is contingent upon the time elapsed since the loss of forest cover,^20^ ^,^ ^21^ and the rate of deforestation also influences vector efficiency.^21^ ^,^ ^22^

In Brazil, the Amazon region accounts for 99% of malaria cases, with approximately 85% attributed to *P. vivax*.^4^ ^,^ ^5^ ^,^ ^14^ The highest malaria burden is observed in rural areas,^14^ ^,^ ^23^ where the past three decades have witnessed a notable increase in deforestation. In Pará, one of the nine states within the Brazilian Amazon, forest devastation is widespread, driven by economic activities such as settlement formation for subsistence farming, the construction of roads and hydroelectric dams, and large-scale deforestation for cattle ranching.^14^ ^,^ ^17^ ^,^ ^23^ ^,^ ^24^ Another significant contributor to environmental changes impacting the malaria incidence is mining.^17^ ^,^ ^24^ Within the Amazon region, malaria is concentrated in select municipalities and categorised by annual parasite incidence (API), ranging from very low to high risk.^25^

This retrospective analysis explores the spatial and temporal patterns of malaria incidence alongside deforestation and its resultant landscape components (composition and configuration) over a 12-year period across all municipalities in Pará State, Brazil. The investigates the potential relationship between environmental changes, particularly deforestation, and malaria incidence in this endemic region.

## MATERIALS AND METHODS

**Fig. 1:**
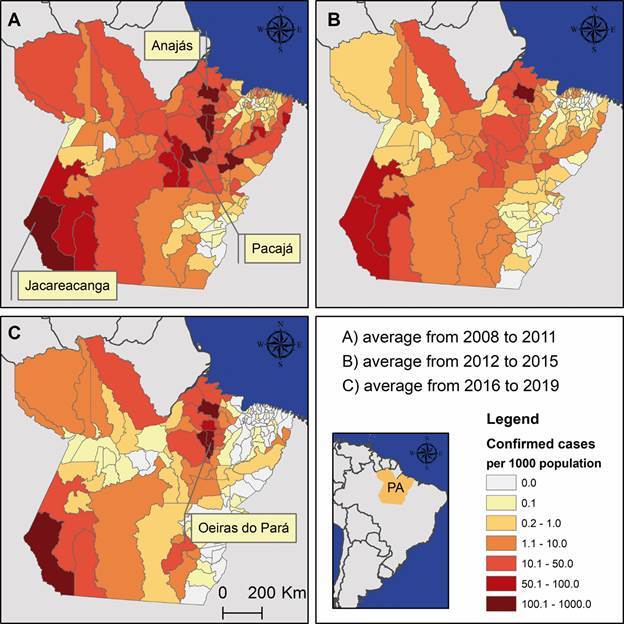
annual malaria incidence rates by municipality in the State of Pará, averaged across study periods: (A) 2008-2011, (B) 2012-2015, and (C) 2016-2019. Source: SIVEP-Malaria. These maps were produced exclusively for this publication using ArcGIS for Desktop (v.10.4.1).

*Study area and data collection* - This study included all 144 municipalities in Pará State, where the annual malaria incidence rates during the study period (2008-2019) ranged from 0 to 750 malaria cases per 1,000 people in the Brazilian Amazon region (Fig. 1). These data were retrieved for each municipality of infection from official malaria data repositories held by the Brazilian Ministry of Health’s malaria surveillance system (SIVEP-Malaria) under the Brazilian Malaria Control Programme.^26^ API, defined as the total number of new malaria cases divided by the total number of examinations per 1,000 people in a given year, was retrieved from SIVEP-Malaria. To evaluate temporal and spatial differences in malaria rates, these API values were averaged across three 4-year periods: 2008-2011, 2012-2015, and 2016-2019. Approval for this study was obtained from the Ethics Committee at the Federal University of Pará (approval number 5.137.483).

**Fig. 2:**
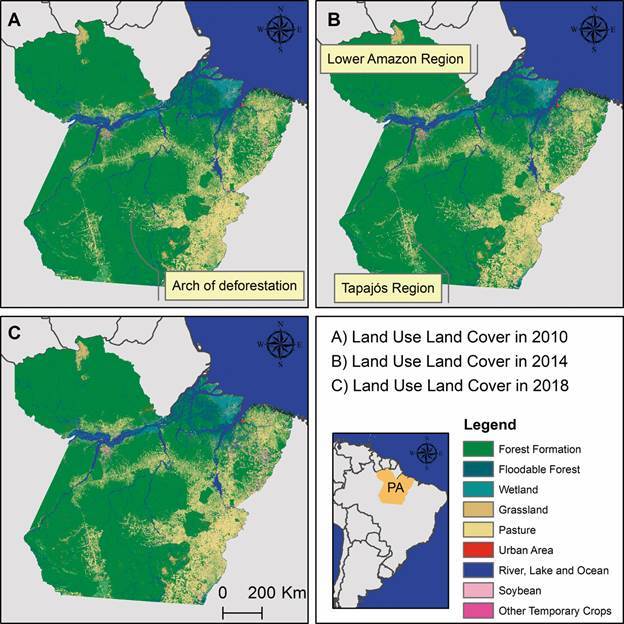
land use and land cover in the State of Pará during selected years, representing forest conservation status at the midpoint of each study period. (A) 2010 for 2008-2011, (B) 2014 for 2012-2015, and (C) 2018 for 2016-2019. Source: MapBiomas v. 9. These maps were produced exclusively for this publication using ArcGIS for Desktop (v.10.4.1).

Deforestation metrics were calculated using publicly available data obtained from MapBiomas v. 9.^27^ The MapBiomas collection, derived from Landsat imagery with a 30-metre spatial resolution, provides annual land use and land cover maps spanning 38 years (1985-2023) (Fig. 2). Deforestation was calculated as loss of forest cover (%) in the previous year, the proportion of forest converted to pasture (%), the proportion converted to urban areas (%), the proportion of retained/restored forest (%), and the number of remaining forest patches (fragmentation). Water levels (%) were included as a control variable. These variables were averaged across the same temporal periods (2008-2011, 2012-2015, and 2016-2019) to explore their association with malaria incidence rates.

**Fig. 3:**
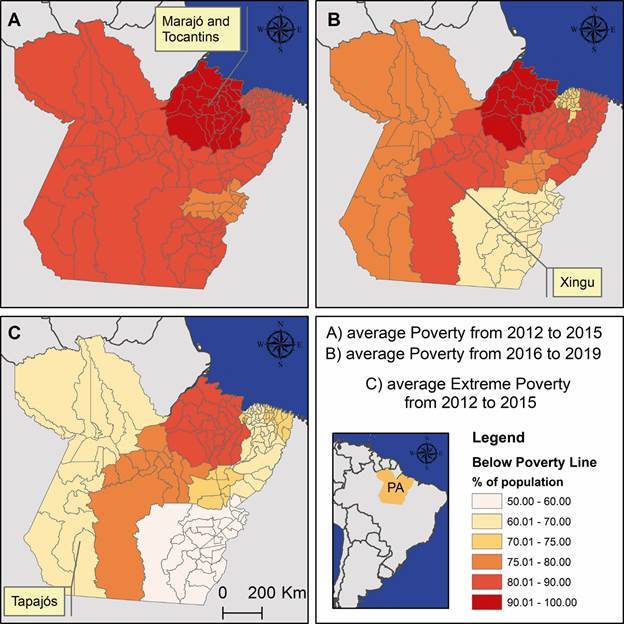
average proportion of municipal population living below poverty or extreme poverty lines in the State of Pará during the available periods. (A) poverty in 2012-2015, (B) poverty in 2016-2019, and (C) extreme poverty in 2012-2015. Source: FAPESPA. These maps were produced exclusively for this publication using ArcGIS for Desktop (v.10.4.1).

Socioeconomic and climate data were obtained from the Fundação Amazônia de Amparo a Estudos e Pesquisas do Pará to support the analysis of the relationship between malaria incidence and deforestation levels.^28^ Poverty and extreme poverty, as defined by the United Nations - poverty being the deprivation of basic human needs and extreme poverty reflecting the inability to meet even basic survival conditions - were mapped using the regions of integration, the official administrative divisions established by the state government of Pará (Fig. 3). Additionally, the Human Development Index for Pará State increased from 0.666 in 2012 to 0.704 in 2019, reflecting positive progress. However, significant challenges remain in ensuring universal healthcare, access to higher education, and equitable income distribution for the entire population. Finally, all municipalities are located within a climatic zone classified as humid equatorial, characterised by two distinct seasons: nine months of humidity and three months of dryness. The average temperature ranges from 25**º**C to 27**º**C.^28^

*Spatial analysis* - A spatial analysis of the relationship between malaria incidence rates and deforestation metrics was conducted for each temporal period (2008-2011, 2012-2015, and 2016-2019) using ArcGIS for Desktop (v.10.4.1) with the Spatial Analyst extension (Esri@ ArcMap™, Redlands, CA, USA). A projected coordinate system, WGS 1984 UTM Zone 22S, was used for all analyses. A spatial weights matrix was constructed using a squared inverse Euclidean distance of 250 km. This distance was selected based on the large territorial size of certain municipalities, such as Altamira or São Félix do Xingu, which has an area comparable to that of several European countries. The spatial weights matrix was employed to capture the spatial structure and connectivity among municipalities in the state. This matrix accounted for territorial area in km^2^ and spatial connectivity (with an average of 40 neighbours, ranging from 1 to 74) and was used to adjust the relationship between malaria incidence rates and deforestation metrics.

First, an exploratory regression of malaria incidence rates for each temporal period was performed against deforestation metrics to test for residual normality and spatial stationarity using the Jarque-Bera and Koenker statistics, multicollinearity through the variance inflation factor, and spatial autocorrelation with the Global Moran Index. These tests informed the selection of the appropriate spatial regression model. Based on the results, a geographically weighted regression (GWR) approach was applied to provide a more nuanced assessment of the relationship between malaria incidence and deforestation across a heterogeneous landscape characterised by varying levels of deforestation and its associated outcomes.

In the GWR approach, each municipality and its neighbouring areas were modelled using local regression analyses for malaria incidence and deforestation metrics, allowing for individual interpretation of the results. As an initial criterion, the standard residuals for each model, ranging from -1.5 to 1.5, were used to assess how well the model fit the actual data and to select the most accurate models for further analysis. In the R programming environment (v. 4.3), the number of significant coefficients for each deforestation metric and temporal period was calculated. The significance of each coefficient was determined by dividing its estimated value by its standard error to obtain a t-value. Coefficients with a t-value of ≥ 1.96 were considered significant (*i.e.*, p < 0.05). The municipalities where significant coefficients representing the relationship between malaria incidence and deforestation metrics were found were mapped to facilitate the interpretation of these results.

## RESULTS

The deforested area in the Amazon region of Pará State over a 12-year period totalled 30,000 km^2^, accounting for approximately 2.5% of the state’s total area of 1,248,000 km^2^ (Fig. 2). The eastern region of Pará experienced the highest deforestation, which extended significantly toward the south. Of particular note is the central part of the state, where a large arch-shaped area, connecting the east to the west, is commonly referred to as the arch of deforestation. In the western region, deforestation is primarily concentrated in two micro-regions: the Lower Amazon and the Tapajós River. While deforestation is widespread throughout Pará, it is particularly concentrated along riverbanks.

The incidence of malaria cases was widespread across nearly all municipalities in the state, but the highest rates were concentrated in a small number of municipalities (Fig. 1). During this period, 679,846 malaria cases were recorded, including 566,014 cases of *P. vivax* (83%), 94,564 cases of *P. falciparum* (14%), 10,319 mixed infections (2%), and 8,949 cases caused by other malaria parasites (1%). Municipalities with malaria incidence rates exceeding 50 cases per 1,000 people were clustered in the regions of integration of Marajó (including Afuá, Anajás, Bagre, and Curralinho), Tapajós (Itaituba, Jacareacanga, and Trairão), Xingu (including Altamira, Anapu, and Pacajá), and Tocantins (Oeiras do Pará). These municipalities can be classified as high-risk areas for malaria transmission.

The regions of integration of Marajó and Tocantins had 90% of their population below the poverty line in 2012-2015 (Fig. 3). During this period, these regions accounted for 90,945 malaria cases, representing 71% of the total malaria cases in the state. The Marajó region remained predominantly below the poverty line in the 2016-2019 period and continued to include municipalities with high-risk malaria transmission. Poverty affected 80% of the population in the integration region of Xingu during both periods (2012-2019), while in the Tapajós region, poverty slightly decreased. In total, 74,249 malaria cases were reported across both regions, accounting for 11% of the total malaria cases in the state during the same period.

**Fig. 4:**
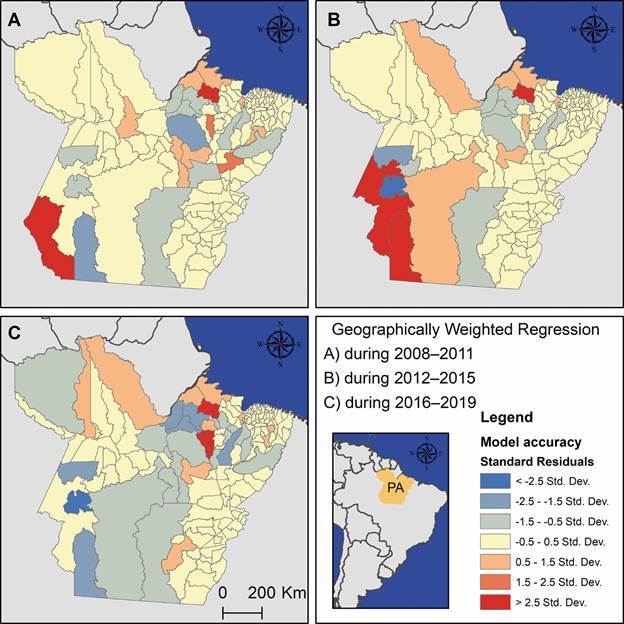
results from the geographically weighted regression (GWR) models, illustrating model accuracy through standard residuals for each study period. (A) 2008-2011, (B) 2012-2015, and (C) 2016-2019. These maps were produced exclusively for this publication using ArcGIS for Desktop (v.10.4.1).

The exploratory regression revealed that the spatial relationship between malaria incidence rates and deforestation metrics resulted in models with non-normal residuals, lack of stationarity, and spatial autocorrelation, but no evidence of multicollinearity. Based on these findings, the GWR approach was selected to account for spatial heterogeneity in the data. This method was particularly useful for modelling the relationship between deforestation and malaria incidence because it is expected to vary across space, allowing for the analysis of localised variations in the estimation of coefficients. It provided a clearer understanding of how these variables interacted across different regions. For instance, the fitted GWR models for each municipality showed varying levels of model accuracy across the state (Fig. 4). The highest accuracy was observed in 138 models for both the 2008-2011 and 2012-2015 periods, and in 133 models for 2016-2019.

**Fig. 5:**
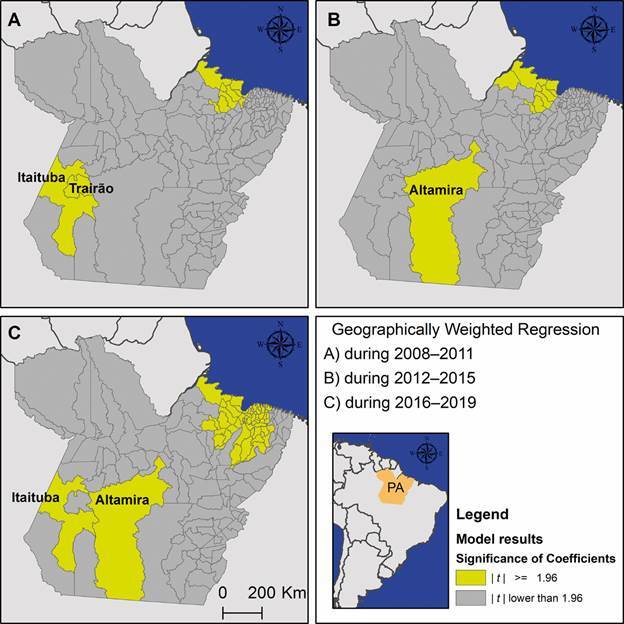
results from the geographically weighted regression (GWR) models, showing significant coefficients by municipality in the State of Pará for each study period. (A) 2008-2011, (B) 2012-2015, and (C) 2016-2019. These maps were produced exclusively for this publication using ArcGIS for Desktop (v.10.4.1).

The GWR models explained 31%, 29%, and 46% of the variance in malaria incidence rates across the three temporal periods, respectively (Table). The analysis identified 12 models with significant coefficients during 2008-2011, all indicating a non-reciprocal (negative) relationship between malaria incidence rates and municipalities with larger areas of pastureland. Additionally, two models from this period revealed a more complex dynamic in the Tapajós region (Fig. 5), where both deforestation and forest cover exhibited negative effects. This finding suggests that in already deforested areas, such as those in Itaituba and Trairão, an increase in forest cover (*e.g.*, through restoration) was associated with a rise in malaria case numbers.

**TABLE:** Results from the geographically weighted regression (GWR) models in Pará State, 2008-2019

Period^*^	R^2^	*n* / *N* ^*a*^	Deforestation indicators and landscape characteristics^*b*^					
			Deforestation	Pasture	Fragmentation	Forest	Urban	Water
2008-2011	31%	12 / 138	2 (-2)	12 (-12)	0	2 (-2)	0	0
2012-2015	29%	10 / 138	0	9 (-9)	1 (+1)	1 (+1)	0	0
2016-2019	46%	56 / 133	2 (+1/-1)	34 (-34)	2 (+2)	56 (+55/-1)	0	0

* All variables, including malaria incidence rates, deforestation metrics, and water levels in the landscape, were averaged for each of the study periods. *a*: in this context, *n* represents the number of municipalities with significant coefficients identified by the GWR model, while *N* denotes the total number of municipalities where the GWR model demonstrated accuracy; *b*: deforestation was calculated as the percentage of forest cover lost in the previous year. Pasture was calculated as the proportion of forest converted to pastureland (%). Fragmentation was measured by the number of remaining forest patches. Forest cover was assessed as the proportion of retained or restored forest (%). Urbanisation was calculated as the proportion of land converted to urban areas (%). Water levels, representing the proportion of surface water in the landscape, were included as a control variable.

During the 2012-2015 period, 10 models demonstrated significant coefficients (Table). Of these, nine indicated a negative relationship between malaria incidence rates and larger areas of pastureland. Additionally, one model highlighted that forested areas with high fragmentation, such as those in Altamira (Fig. 5), were more likely to experience elevated malaria incidence rates.

During the 2016-2019 period, 56 models had significant coefficients (Table). Among these, 34 indicated a negative relationship between malaria incidence rates and pasture, consistent with findings from earlier periods. Additionally, 55 models demonstrated a positive relationship between forest cover and higher malaria incidence rates. Two models revealed more complex dynamics in the Tapajós and Xingu regions (Fig. 5). In Itaituba, increased deforestation and greater forest fragmentation in areas with reduced forest cover were linked to higher malaria incidence rates. Similarly, in Altamira, forested areas undergoing recovery and exhibiting high levels of forest fragmentation also showed elevated malaria incidence rates.

## DISCUSSION

This study examined the incidence of malaria in the Brazilian Amazon rainforest, focusing on endemic areas with varying levels of accumulated deforestation. In the high-transmission eastern area of Pará, Anajás on Marajó Island exhibits minimal deforestation, whereas Oeiras do Pará and Pacajá have undergone extensive deforestation. In western Pará, where recent deforestation is prevalent, Itaituba and Jacareacanga report elevated malaria transmission. These five municipalities, selected for their historical significance, accounted for nearly 50% of Pará’s malaria cases from 2008 to 2019.

Since the 1970s, following the inauguration of the Belém-Brasília and Transamazônica highways, significant human migration driven by economic opportunities has sustained high levels of both deforestation and malaria transmission in the Amazon region. Determinants of deforestation in Pará include extensive agricultural projects, cattle ranching, and the creation of large reservoirs for hydroelectric dams.^7^ ^,^ ^14^ ^,^ ^17^ ^,^ ^23^ The GWR models presented here show that water levels in the landscape are not associated with contemporary malaria incidence rates, despite the well-documented explosive rise in malaria cases linked to the construction of the Tucuruí hydroelectric plant between 1975 and 1984. This period led to a high transmission risk and an outbreak of the disease upon completion of the project.^7^ Additionally, the GWR models indicate that pasturelands now have a negative effect on malaria transmission risk, although it has been documented that at the onset of such activities, malaria can emerge as an endemic disease, closely linked to changes that increase exposure to malaria vectors.^16^ ^,^ ^21^ ^,^ ^22^ ^,^ ^29^ This result supports the unimodal relationship between malaria risk and the landscape in the Amazon, demonstrating higher risk in areas where forests are being converted and lower risk in regions that are already heavily degraded.^9^ ^,^ ^21^ ^,^ ^29^ ^,^ ^30^

The housing conditions along riverbanks in the Marajó region, particularly in Anajás, which are associated with high levels of poverty, contribute to increased exposure to malaria vectors. During the past two decades, Anajás has consistently been classified as a high-risk area for malaria.^25^ Additionally, the Tapajós and Xingu regions, particularly Itaituba and Altamira - some of the most remote areas in Pará - have experienced recent population growth driven by migration, alongside local economic activities such as mining (more frequent in Itaituba) and agricultural land use.^16^ ^,^ ^24^ The GWR models reveal that these regions are characterised by high forest cover (> 50%) but are experiencing forest fragmentation and ongoing modifications in forest cover, including both forest loss and restoration. Forest loss and restoration are part of a broader process of landscape alteration, driven by the conversion of forests into economically profitable land for rotational cattle ranching.^21^ ^,^ ^29^ This rotation allows the land to remain productive for cattle farming while abandoned plots regenerate, creating new habitats for malaria vectors, particularly *Nyssorhynchus darlingi* (formerly *Anopheles darlingi*), a species considered an intermediate forest disturbance specialist.^20^ ^,^ ^30^ As a result, malaria risk is heightened due to these activities, emphasising the need for intensified surveillance efforts to reduce its incidence in the Tapajós region, which has been recently impacted by deforestation.

Malaria incidence rates declined over the three study periods, although some municipalities exhibited variable transmission patterns. For instance, Oeiras do Pará had an average malaria incidence of 258 per 1,000 people during 2008-2011, which decreased to 49 in 2012-2015 but rose again to 169 in 2016-2019. This variation is common across all states of the Brazilian Amazon.^6^ ^,^ ^31^ ^,^ ^32^ These fluctuations are driven not only by economic activities, as discussed earlier, but also by changes in malaria control efforts.^24^ ^,^ ^25^ ^,^ ^33^ Malaria control measures - including prompt diagnostics, adequate treatment, and vector control through insecticide-treated bed nets - were implemented in all municipalities as part of the National Malaria Control Programme, in which Pará State is a key participant.^2^ ^,^ ^3^ ^,^ ^26^ However, even with these control measures, transmission rates may stabilise due to specific challenges, such as the presence of asymptomatic reservoirs carrying *P. vivax* and outdoor biting by malaria vectors such as *Ny. darlingi*.^14^ ^,^ ^32^ ^,^ ^34^ ^-^ ^37^ In a simulated scenario, if all or any of these control measures were abruptly suspended, the incidence of malaria could increase exponentially in municipalities with malaria incidence rates of 10 per 1,000 people or higher.

The complex relationship between deforestation and malaria incidence^8^ ^,^ ^12^ ^,^ ^33^ ^,^ ^35^ complicates its interpretation, with studies showing varying results.^9^ ^,^ ^11^ ^,^ ^20^ ^,^ ^38^ Generally, in the Amazon, initial deforestation in newly settled forest areas tends to increase malaria risk up to a certain threshold of forest cover and social development.^22^ ^,^ ^29^ ^,^ ^36^ Beyond this threshold, as deforestation progresses, the risk of malaria may decrease.^30^ ^,^ ^33^ The GWR models provided two key insights: first, large pasturelands are not conducive to malaria transmission, even if deforestation continues; second, regions with high forest cover under pressure from forest loss and fragmentation are likely to experience higher malaria transmission. These findings align with previous studies^20^ ^,^ ^21^ ^,^ ^29^ ^,^ ^30^ ^,^ ^33^ that emphasise the intricate relationship between land-use changes and malaria risk.

While ongoing discussions persist, a recent investigation^39^ identified Pará State as having the highest cumulative deforestation between 2003 and 2022. This analysis revealed that four of the top five most heavily deforested indigenous territories and three of the five most impacted conservation units were located within Pará. Additionally, the study^39^ found that a 1% increase in deforestation, with a 1-month lag, corresponded to a 6% rise in malaria cases at the municipal level. Pará is also home to one of the highest diversities of malaria vector species.^40^ Given the vast ecological, sociodemographic, economic, and epidemiological diversity of the Brazilian Amazon, it is crucial to dissect the multifaceted drivers of deforestation and subsequent malaria transmission. Only by understanding these dynamics can actionable insights support Brazil’s ambitious malaria elimination efforts.^32^

This study provides valuable insights into the links between deforestation and malaria in Pará State using robust methods and accessible data. However, it is important to acknowledge the limitations of this approach. Aggregating data over multiple years and conducting analyses at the municipal level may obscure localised or short-term variations. While this approach offers a broad perspective, it may overlook finer-scale dynamics of malaria transmission patterns.

*In conclusions* - Pará State continues to face challenges in malaria elimination, with incidence closely linked to areas of significant forest loss and fragmentation. These findings highlight how changes in forest composition influence malaria risk, emphasising the need for tailored strategies that integrate environmental and socioeconomic factors to support Brazil’s 2035 malaria elimination plan.
